# (*E*)-3-(4-Chloro­phen­yl)-1-(4-fluoro­phenyl)­prop-2-en-1-one

**DOI:** 10.1107/S1600536812004230

**Published:** 2012-02-10

**Authors:** Hoong-Kun Fun, Tze Shyang Chia, M. Sapnakumari, B. Narayana, B. K. Sarojini

**Affiliations:** a X-ray Crystallography Unit, School of Physics, Universiti Sains Malaysia, 11800 USM, Penang, Malaysia; b Department of Studies in Chemistry, Mangalore University, Mangalagangotri 574 199, India; c Department of Chemistry, P. A. College of Engineering, Nadupadavu, Mangalore 574 153, India

## Abstract

In the title compound, C_15_H_10_ClFO, the fluoro-substituted benzene ring forms a dihedral angle of 44.41 (6)° with the chloro-substituted benzene ring. The only significant directional bonds in the crystal are weak C—H⋯π inter­actions.

## Related literature

For related structures and background to chalcone derivatives, see: Fun, Loh *et al.* (2011 ▶); Fun, Arshad *et al.* (2011*a* ▶,*b* ▶). For the stability of the temperature controller used for data collection, see: Cosier & Glazer (1986 ▶).
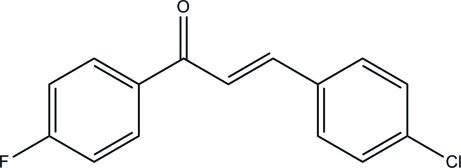


## Experimental

### 

#### Crystal data

C_15_H_10_ClFO
*M* *_r_* = 260.68
Triclinic, 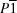

*a* = 5.8875 (3) Å
*b* = 7.4926 (3) Å
*c* = 13.6022 (6) Å
α = 80.351 (1)°
β = 85.483 (1)°
γ = 83.545 (1)°
*V* = 586.69 (5) Å^3^
*Z* = 2
Mo *K*α radiation
μ = 0.32 mm^−1^
*T* = 100 K
0.38 × 0.25 × 0.10 mm

#### Data collection

Bruker APEX DUO CCD diffractometer
Absorption correction: multi-scan (*SADABS*; Bruker, 2009 ▶) *T*_min_ = 0.887, *T*_max_ = 0.970
12071 measured reflections
3057 independent reflections
2785 reflections with *I* > 2σ(*I*)
*R*_int_ = 0.021

#### Refinement

*R*[*F* ^2^ > 2σ(*F* ^2^)] = 0.032
*wR*(*F* ^2^) = 0.086
*S* = 1.07
3057 reflections
163 parameters
H-atom parameters constrained
Δρ_max_ = 0.45 e Å^−3^
Δρ_min_ = −0.25 e Å^−3^

### 

Data collection: *APEX2* (Bruker, 2009 ▶); cell refinement: *SAINT* (Bruker, 2009 ▶); data reduction: *SAINT*; program(s) used to solve structure: *SHELXTL* (Sheldrick, 2008 ▶); program(s) used to refine structure: *SHELXTL*; molecular graphics: *SHELXTL*; software used to prepare material for publication: *SHELXTL* and *PLATON* (Spek, 2009 ▶).

## Supplementary Material

Crystal structure: contains datablock(s) global, I. DOI: 10.1107/S1600536812004230/hb6622sup1.cif

Structure factors: contains datablock(s) I. DOI: 10.1107/S1600536812004230/hb6622Isup2.hkl

Supplementary material file. DOI: 10.1107/S1600536812004230/hb6622Isup3.cml

Additional supplementary materials:  crystallographic information; 3D view; checkCIF report

## Figures and Tables

**Table 1:** Hydrogen-bond geometry (Å, °) *Cg*2 is the centroid of the C10–C15 benzene ring.

*D*—H⋯*A*	*D*—H	H⋯*A*	*D*⋯*A*	*D*—H⋯*A*
C2—H2*A*⋯*Cg*2^i^	0.93	2.85	3.4390 (13)	122
C5—H5*A*⋯*Cg*2^ii^	0.93	2.85	3.3989 (13)	119

Symmetry codes: (i) 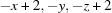; (ii) 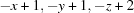.

**Fig. 1.**
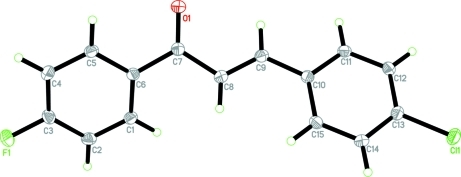
The molecular structure of the title compound with 50% probability displacement ellipsoids.

## supplementary crystallographic information

### Comment

In continuation of our work on the synthesis and structures of chalcone derivatives (Fun, Arshad *et al.*, 2011*a*,*b*; Fun, Loh *et al.*, 2011), the title compound was prepared and its crystal structure is reported.

The molecular structure of the title compound is shown in Fig. 1. The least-squares plane of the fluoro-substituted benzene ring (C1–C6) makes a dihedral angle of 44.41 (6)° with the least-squares plane of the chloro-substituted benzene ring (C10–C15). Bond lengths are comparable to those in related structures (Fun, Arshad *et al.*, 2011*a*,*b*; Fun, Loh *et al.*, 2011).

In the crystal structure, no significant intermolecular hydrogen bonds are observed. The crystal structure features were C—H···π interactions (Table 1), involving the centroid of C10–C15 benzene ring.

### Experimental

To a mixture of 4-fluoroacetophenone (1.38 g, 0.01 mol) and 4-chlorobenzaldehyde (1.41 g, 0.01 mol) in ethanol (100 ml), 15 ml of 10% sodium hydroxide solution was added and stirred at 0–5 °C for 3 h. The precipitate formed was collected by filtration and purified by recrystallization from ethanol. Colourless blocks were grown from toluene as solvent by the slow evaporation method (*m.p.*: 405–407 K).

### Refinement

All H atoms were positioned geometrically [C—H = 0.93 Å] and refined using a riding model with *U*_iso_(H) = 1.2 *U*_eq_(C). An outlier (-3 - 1 7) was omitted.

### Crystal data

**Table:**

C_15_H_10_ClFO	*Z* = 2
*M**_r_* = 260.68	*F*(000) = 268
Triclinic, *P*1	*D*_x_ = 1.476 Mg m^−^^3^
Hall symbol: -P 1	Mo *K*α radiation, λ = 0.71073 Å
*a* = 5.8875 (3) Å	Cell parameters from 7331 reflections
*b* = 7.4926 (3) Å	θ = 3.0–32.5°
*c* = 13.6022 (6) Å	µ = 0.32 mm^−^^1^
α = 80.351 (1)°	*T* = 100 K
β = 85.483 (1)°	Block, colourless
γ = 83.545 (1)°	0.38 × 0.25 × 0.10 mm
*V* = 586.69 (5) Å^3^	

### Data collection

**Table:**

Bruker APEX DUO CCD diffractometer	3057 independent reflections
Radiation source: fine-focus sealed tube	2785 reflections with *I* > 2σ(*I*)
Graphite monochromator	*R*_int_ = 0.021
φ and ω scans	θ_max_ = 29.0°, θ_min_ = 1.5°
Absorption correction: multi-scan (*SADABS*; Bruker, 2009)	*h* = −8→8
*T*_min_ = 0.887, *T*_max_ = 0.970	*k* = −9→10
12071 measured reflections	*l* = −18→18

### Refinement

**Table:**

Refinement on *F*^2^	Primary atom site location: structure-invariant direct methods
Least-squares matrix: full	Secondary atom site location: difference Fourier map
*R*[*F*^2^ > 2σ(*F*^2^)] = 0.032	Hydrogen site location: inferred from neighbouring sites
*wR*(*F*^2^) = 0.086	H-atom parameters constrained
*S* = 1.07	*w* = 1/[σ^2^(*F*_o_^2^) + (0.040*P*)^2^ + 0.2844*P*] where *P* = (*F*_o_^2^ + 2*F*_c_^2^)/3
3057 reflections	(Δ/σ)_max_ = 0.001
163 parameters	Δρ_max_ = 0.45 e Å^−^^3^
0 restraints	Δρ_min_ = −0.25 e Å^−^^3^

### Special details

**Table:**

Experimental. The crystal was placed in the cold stream of an Oxford Cryosystems Cobra open-flow nitrogen cryostat (Cosier & Glazer, 1986) operating at 100.0 (1) K.
Geometry. All e.s.d.'s (except the e.s.d. in the dihedral angle between two l.s. planes) are estimated using the full covariance matrix. The cell e.s.d.'s are taken into account individually in the estimation of e.s.d.'s in distances, angles and torsion angles; correlations between e.s.d.'s in cell parameters are only used when they are defined by crystal symmetry. An approximate (isotropic) treatment of cell e.s.d.'s is used for estimating e.s.d.'s involving l.s. planes.
Refinement. Refinement of *F*^2^ against ALL reflections. The weighted *R*-factor *wR* and goodness of fit *S* are based on *F*^2^, conventional *R*-factors *R* are based on *F*, with *F* set to zero for negative *F*^2^. The threshold expression of *F*^2^ > σ(*F*^2^) is used only for calculating *R*-factors(gt) *etc*. and is not relevant to the choice of reflections for refinement. *R*-factors based on *F*^2^ are statistically about twice as large as those based on *F*, and *R*- factors based on ALL data will be even larger.

### Fractional atomic coordinates and isotropic or equivalent isotropic displacement parameters (Å^2^)

**Table:**

	*x*	*y*	*z*	*U*_iso_*/*U*_eq_	
Cl1	1.24305 (5)	0.70656 (4)	0.56678 (2)	0.02277 (9)	
F1	0.67229 (14)	−0.16353 (11)	1.43564 (6)	0.02659 (18)	
O1	0.28078 (15)	0.20326 (13)	1.02611 (7)	0.02103 (19)	
C1	0.75843 (19)	0.01108 (16)	1.17275 (9)	0.0167 (2)	
H1A	0.8750	0.0160	1.1224	0.020*	
C2	0.8030 (2)	−0.07852 (16)	1.26851 (9)	0.0181 (2)	
H2A	0.9476	−0.1363	1.2827	0.022*	
C3	0.6274 (2)	−0.07948 (16)	1.34189 (9)	0.0180 (2)	
C4	0.4079 (2)	0.00187 (16)	1.32499 (9)	0.0187 (2)	
H4A	0.2937	−0.0005	1.3764	0.022*	
C5	0.36448 (19)	0.08690 (16)	1.22882 (9)	0.0166 (2)	
H5A	0.2177	0.1402	1.2149	0.020*	
C6	0.53854 (19)	0.09366 (15)	1.15221 (8)	0.0145 (2)	
C7	0.4793 (2)	0.18765 (16)	1.05049 (9)	0.0159 (2)	
C8	0.6648 (2)	0.26517 (16)	0.98177 (9)	0.0171 (2)	
H8A	0.8077	0.2703	1.0053	0.021*	
C9	0.62591 (19)	0.32760 (15)	0.88590 (9)	0.0159 (2)	
H9A	0.4837	0.3114	0.8654	0.019*	
C10	0.78251 (19)	0.41862 (15)	0.80970 (8)	0.0146 (2)	
C11	0.72368 (19)	0.45188 (15)	0.70992 (9)	0.0157 (2)	
H11A	0.5880	0.4140	0.6939	0.019*	
C12	0.8636 (2)	0.54020 (16)	0.63434 (9)	0.0166 (2)	
H12A	0.8234	0.5610	0.5683	0.020*	
C13	1.0645 (2)	0.59650 (15)	0.65976 (8)	0.0163 (2)	
C14	1.12740 (19)	0.56716 (15)	0.75820 (9)	0.0161 (2)	
H14A	1.2621	0.6071	0.7738	0.019*	
C15	0.98717 (19)	0.47788 (15)	0.83265 (8)	0.0158 (2)	
H15A	1.0290	0.4570	0.8984	0.019*	

### Atomic displacement parameters (Å^2^)

**Table:**

	*U*^11^	*U*^22^	*U*^33^	*U*^12^	*U*^13^	*U*^23^
Cl1	0.02360 (16)	0.02747 (16)	0.01765 (15)	−0.01091 (11)	0.00178 (11)	−0.00064 (11)
F1	0.0263 (4)	0.0319 (4)	0.0177 (4)	−0.0017 (3)	−0.0043 (3)	0.0076 (3)
O1	0.0149 (4)	0.0287 (5)	0.0188 (4)	−0.0027 (3)	−0.0030 (3)	−0.0005 (3)
C1	0.0139 (5)	0.0187 (5)	0.0175 (5)	−0.0019 (4)	0.0003 (4)	−0.0034 (4)
C2	0.0144 (5)	0.0178 (5)	0.0216 (6)	0.0002 (4)	−0.0031 (4)	−0.0015 (4)
C3	0.0205 (5)	0.0171 (5)	0.0158 (5)	−0.0035 (4)	−0.0035 (4)	0.0014 (4)
C4	0.0168 (5)	0.0209 (5)	0.0173 (5)	−0.0031 (4)	0.0017 (4)	−0.0008 (4)
C5	0.0129 (5)	0.0175 (5)	0.0189 (5)	−0.0014 (4)	−0.0006 (4)	−0.0016 (4)
C6	0.0145 (5)	0.0145 (5)	0.0146 (5)	−0.0027 (4)	−0.0015 (4)	−0.0019 (4)
C7	0.0155 (5)	0.0166 (5)	0.0155 (5)	−0.0024 (4)	−0.0012 (4)	−0.0024 (4)
C8	0.0142 (5)	0.0197 (5)	0.0175 (5)	−0.0034 (4)	−0.0011 (4)	−0.0021 (4)
C9	0.0139 (5)	0.0158 (5)	0.0181 (5)	−0.0016 (4)	−0.0008 (4)	−0.0024 (4)
C10	0.0139 (5)	0.0141 (5)	0.0155 (5)	−0.0003 (4)	−0.0009 (4)	−0.0019 (4)
C11	0.0138 (5)	0.0166 (5)	0.0171 (5)	−0.0016 (4)	−0.0025 (4)	−0.0026 (4)
C12	0.0174 (5)	0.0181 (5)	0.0142 (5)	−0.0013 (4)	−0.0026 (4)	−0.0016 (4)
C13	0.0163 (5)	0.0158 (5)	0.0161 (5)	−0.0018 (4)	0.0014 (4)	−0.0014 (4)
C14	0.0136 (5)	0.0169 (5)	0.0183 (5)	−0.0021 (4)	−0.0019 (4)	−0.0038 (4)
C15	0.0156 (5)	0.0175 (5)	0.0143 (5)	−0.0002 (4)	−0.0024 (4)	−0.0023 (4)

### Geometric parameters (Å, º)

**Table:**

Cl1—C13	1.7379 (12)	C8—C9	1.3380 (16)
F1—C3	1.3560 (13)	C8—H8A	0.9300
O1—C7	1.2279 (14)	C9—C10	1.4640 (15)
C1—C2	1.3919 (16)	C9—H9A	0.9300
C1—C6	1.3985 (15)	C10—C11	1.4020 (15)
C1—H1A	0.9300	C10—C15	1.4044 (16)
C2—C3	1.3790 (17)	C11—C12	1.3909 (16)
C2—H2A	0.9300	C11—H11A	0.9300
C3—C4	1.3845 (17)	C12—C13	1.3862 (16)
C4—C5	1.3853 (16)	C12—H12A	0.9300
C4—H4A	0.9300	C13—C14	1.3927 (16)
C5—C6	1.4016 (15)	C14—C15	1.3856 (16)
C5—H5A	0.9300	C14—H14A	0.9300
C6—C7	1.4919 (15)	C15—H15A	0.9300
C7—C8	1.4844 (16)		
			
C2—C1—C6	120.06 (10)	C7—C8—H8A	120.1
C2—C1—H1A	120.0	C8—C9—C10	127.19 (11)
C6—C1—H1A	120.0	C8—C9—H9A	116.4
C3—C2—C1	118.38 (11)	C10—C9—H9A	116.4
C3—C2—H2A	120.8	C11—C10—C15	118.49 (10)
C1—C2—H2A	120.8	C11—C10—C9	118.77 (10)
F1—C3—C2	118.32 (11)	C15—C10—C9	122.72 (10)
F1—C3—C4	118.26 (10)	C12—C11—C10	121.49 (10)
C2—C3—C4	123.42 (11)	C12—C11—H11A	119.3
C3—C4—C5	117.61 (11)	C10—C11—H11A	119.3
C3—C4—H4A	121.2	C13—C12—C11	118.42 (10)
C5—C4—H4A	121.2	C13—C12—H12A	120.8
C4—C5—C6	120.94 (11)	C11—C12—H12A	120.8
C4—C5—H5A	119.5	C12—C13—C14	121.65 (11)
C6—C5—H5A	119.5	C12—C13—Cl1	119.39 (9)
C1—C6—C5	119.56 (10)	C14—C13—Cl1	118.95 (9)
C1—C6—C7	122.44 (10)	C15—C14—C13	119.32 (10)
C5—C6—C7	118.00 (10)	C15—C14—H14A	120.3
O1—C7—C8	121.55 (10)	C13—C14—H14A	120.3
O1—C7—C6	120.21 (10)	C14—C15—C10	120.63 (10)
C8—C7—C6	118.22 (10)	C14—C15—H15A	119.7
C9—C8—C7	119.82 (10)	C10—C15—H15A	119.7
C9—C8—H8A	120.1		
			
C6—C1—C2—C3	−1.50 (17)	C6—C7—C8—C9	170.70 (11)
C1—C2—C3—F1	−178.63 (10)	C7—C8—C9—C10	175.68 (11)
C1—C2—C3—C4	0.96 (18)	C8—C9—C10—C11	171.25 (11)
F1—C3—C4—C5	−179.88 (10)	C8—C9—C10—C15	−10.24 (19)
C2—C3—C4—C5	0.53 (18)	C15—C10—C11—C12	0.45 (17)
C3—C4—C5—C6	−1.49 (18)	C9—C10—C11—C12	179.03 (10)
C2—C1—C6—C5	0.58 (17)	C10—C11—C12—C13	−0.35 (17)
C2—C1—C6—C7	−178.48 (11)	C11—C12—C13—C14	−0.20 (17)
C4—C5—C6—C1	0.96 (17)	C11—C12—C13—Cl1	179.80 (9)
C4—C5—C6—C7	−179.94 (10)	C12—C13—C14—C15	0.65 (17)
C1—C6—C7—O1	155.13 (12)	Cl1—C13—C14—C15	−179.35 (9)
C5—C6—C7—O1	−23.93 (17)	C13—C14—C15—C10	−0.54 (17)
C1—C6—C7—C8	−26.56 (16)	C11—C10—C15—C14	0.00 (17)
C5—C6—C7—C8	154.38 (11)	C9—C10—C15—C14	−178.52 (10)
O1—C7—C8—C9	−11.02 (18)		

### Hydrogen-bond geometry (Å, º)

Cg2 is the centroid of the C10–C15 benzene ring.

**Table:**

*D*—H···*A*	*D*—H	H···*A*	*D*···*A*	*D*—H···*A*
C2—H2*A*···*Cg*2^i^	0.93	2.85	3.4390 (13)	122
C5—H5*A*···*Cg*2^ii^	0.93	2.85	3.3989 (13)	119

Symmetry codes: (i) −*x*+2, −*y*, −*z*+2; (ii) −*x*+1, −*y*+1, −*z*+2.
